# Mutagenesis by AID: Being in the Right Place at the Right Time

**DOI:** 10.1371/journal.pgen.1005489

**Published:** 2015-09-10

**Authors:** Cristina Rada

**Affiliations:** MRC Laboratory of Molecular Biology, Cambridge, United Kingdom; CABIMER, Universidad de Sevilla, SPAIN

Deamination of cytosine is a common consequence of the natural hydrolytic decay of DNA. However, it is also part of a mutagenesis programme in immune cells, initiated by activation-induced cytidine deaminase (AID) acting on the genes that encode antibodies. Cytosine deamination by AID results in a staggering number of localised point mutations. This is achieved within a few cell divisions despite the rather innocuous nature of base change in the DNA, deoxy-uracil, which is efficiently removed by the uracil-DNA glycosylase (UNG). Highly abundant during S phase, the main role of UNG is to remove uracil misincorporated during DNA replication, but it can act at other times, both on double and single stranded DNA (dsDNA and ssDNA, respectively). Cells have a back up glycosylase, SMUG1 [1], that removes uracil from double stranded DNA, a pathway ideally suited to faithfully repair deaminated cytosines outside replication. It has been quite a mystery why repair of uracils induced by AID is mostly mutagenic; a favoured hypothesis was that uracil excision at the wrong time during the cell cycle would prevent faithful repair. The work by Le and Maizels [2] on this issue goes some way to resolving this question.

AID is a powerful mutator of single stranded DNA in B cells. Its activity can be misdirected to other parts of the genome, leading to translocations and oncogenic transformation in many B cell malignancies. It is also responsible for clustered kataegic mutations, the telltale of indiscriminate deaminase activity on ssDNA [3] found in Chronic Lymphocytic Leukemias and other cancers [4,5]. It is therefore not surprising that under normal circumstances AID entry into the cell nucleus is tightly regulated and, once there, its abundance is limited. The key mechanism regulating AID abundance is through nuclear degradation and, in order to balance this, AID is rapidly exported to the cytoplasm where it resides as part of a stable complex [6]. Indeed, loss of AID’s C-terminal nuclear export signal, a mutation observed in immunodeficiency patients, leads to rapid degradation of the protein as it cannot be translocated back to the cytosol.

**Fig 1:**
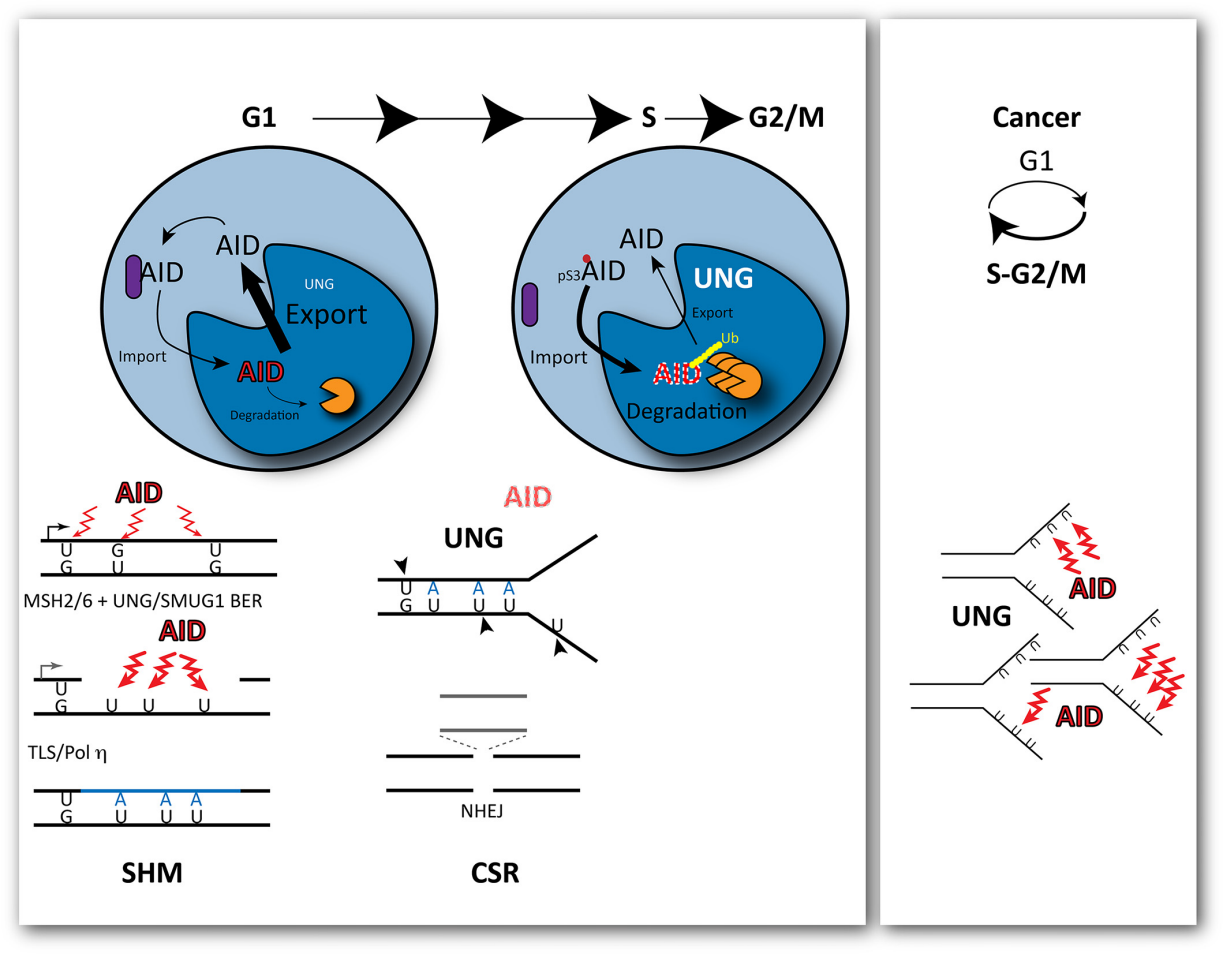
Outcomes of cytosine deamination during the cell cycle. AID is selectively recruited to the immunoglobulin locus by transcription during G1. Its mutagenic activity is restricted to G1 during the cell cycle, with rapid nuclear degradation during S phase. This is modulated by phosphorylation of AID at Ser3, which promotes its rapid degradation in the cell nucleus. As a consequence, cytosine deamination-resulting uracils opposite guanines are processed before replication by base excision repair and non-canonical mismatch repair, creating single stranded gaps in the DNA and further localised substrates for deamination. At the transition to S phase, the levels of UNG, the uracil glycosylase that removes uracils, are increased leading to double-strand breaks and deletions that promote class switching though the non-homologous end joining of the broken ends. In cancer cells, cell-cycle deregulation can expose single stranded DNA during replication to the activity of AID, leading to clustered mutations.

Thus, the mutagenic activity of AID depends on its ability to overcome faithful uracil repair but also to remain in the nucleus of B cells. In an elegant study, Le and Maizels [2] have illuminated important aspects of how this is accomplished by manipulating the subcellular localisation of AID at different times of the cell cycle. By fusing AID to cell-cycle regulated "degrons," they limited the levels of AID to mainly G1 or S-G2/M and show that deamination by AID is only mutagenic during G1 (Fig 1). This had been intimated by the Reynaud group in 2002 [7] based on mutation emergence post-AID induction and, more recently, by the Jolly group, by inhibiting UNG in G1 or S-G2/M [8] using a strategy similar to Le and Maizels [2]. However, neither study separated AID activity from processing of the deaminated C or explained how AID activity was restricted to G1. The new study by Le and Maizels fully explains these results by demonstrating that the enforced presence of AID in the nucleus is toxic in S phase (with the cellular response leading to its rapid degradation), whereas it is tolerated during G1, when it enhances hypermutation and class switching [2]. The study goes further in providing some mechanistic insight as to how phosphorylation of AID modulates its cell cycle related stability.

Mutagenesis induced by AID results from a plethora of misfortunes, from simple miscoding by uracil/abasic sites to mismatch-induced error-prone repair or the generation of double strand breaks (DSBs) resolved with loss of genetic information by the non-homologous end joining (NHEJ) pathway. All these events can result from engaging DNA repair at the wrong time, during G1 (Fig 1). Recruitment of the Mismatch Repair (MMR) factor MSH2 to U:G mismatches in dsDNA prior to replication in the presence of nearby uracils that are substrates of Base Excision Repair (BER), which leads to ssDNA gaps [9] (a further substrate of deamination and uracil excision) and, eventually, translesion DNA synthesis by Polη. Increased UNG levels in early S phase facilitate uracil excision in ss and dsDNA, leading to breaks before the homologous recombination pathway has a chance to act and to NHEJ-mediated deletions.

With AID activity restricted to G1, it follows that secondary deaminations on the ssDNA generated during MMR of the initial U:G mispairs could be perpetuated by Polη during gap filling as U:A pairs. These uracils would no longer be the substrate of MMR or a preferred substrate for SMUG1 (preventing futile rounds of additional MMR or BER), but would offer additional substrates for UNG. This scenario also obviates a requirement for persistent abasic sites, which are intrinsically chemically unstable, for the generation of transversions at G:C pairs or of breaks. Indeed, the strict association of cell division with DSBs required for class switching could just reflect a requirement for high levels of UNG activity on closely spaced U:G and U:A pairs.

Le and Maizels go on to show that the abundance of AID is modulated by phosphorylation at Ser3, resulting in enhanced degradation in G1 [2]. This suggests that the balance between nuclear import and export of AID is cell-cycle regulated. AID is preferentially recruited to the immunoglobulin locus by transcription, which takes place mostly during G1. Thus, its activity leads to localized mutagenesis when its presence in the cell nucleus can be tolerated. During S phase, when ssDNA is abundant, AID's presence in the nucleus would lead to catastrophic global mutagenesis; therefore, it is actively degraded, exported, and kept away in a cytosolic complex [10]. Questions remain regarding how phosphorylation itself is regulated, and how exactly it modulates AID abundance: does it increase active import, does it reduce export, or does it release cytosolic retention? Alternatively, phosphorylation could affect the ubiquitin dependent and/or independent degradation of nuclear AID.

In the meantime, Le and Maizels findings also offer an immediate practical application, expanding the potential use of modified or enhanced AID enzymes as a biotechnology tool to evolve antibodies in vitro by manipulating the toxicity of AID in cells and targeting its activity to the time-window during which it can best do its job [2].
